# Terahertz spectroscopy on Faraday and Kerr rotations in a quantum anomalous Hall state

**DOI:** 10.1038/ncomms12245

**Published:** 2016-07-20

**Authors:** Ken N. Okada, Youtarou Takahashi, Masataka Mogi, Ryutaro Yoshimi, Atsushi Tsukazaki, Kei S. Takahashi, Naoki Ogawa, Masashi Kawasaki, Yoshinori Tokura

**Affiliations:** 1 RIKEN Center for Emergent Matter Science (CEMS), Wako 351-0198, Japan; 2 Department of Applied Physics and Quantum Phase Electronics Center (QPEC), University of Tokyo, Tokyo 113-8656, Japan; 3 PRESTO, Japan Science and Technology Agency (JST), Chiyoda-ku, Tokyo 102-0075, Japan; 4 Institute for Materials Research, Tohoku University, Sendai 980-8577, Japan

## Abstract

Electrodynamic responses from three-dimensional topological insulators are characterized by the universal magnetoelectric term constituent of the Lagrangian formalism. The quantized magnetoelectric coupling, which is generally referred to as topological magnetoelectric effect, has been predicted to induce exotic phenomena including the universal low-energy magneto-optical effects. Here we report the experimental indication of the topological magnetoelectric effect, which is exemplified by magneto-optical Faraday and Kerr rotations in the quantum anomalous Hall states of magnetic topological insulator surfaces by terahertz magneto-optics. The universal relation composed of the observed Faraday and Kerr rotation angles but not of any material parameters (for example, dielectric constant and magnetic susceptibility) well exhibits the trajectory towards the fine structure constant in the quantized limit.

## Abstract (web summary)

Quantized magnetoelectric coupling, which induces exotic magneto-optical phenomena, awaits to be evidenced in topological insulators. Here, Okada *et al*. demonstrate Faraday and Kerr rotations of magnetic topological insulator surfaces in quantum anomalous Hall states by terahertz magneto-optics, indicating topological magnetoelectric effect.

## Graphical abstract

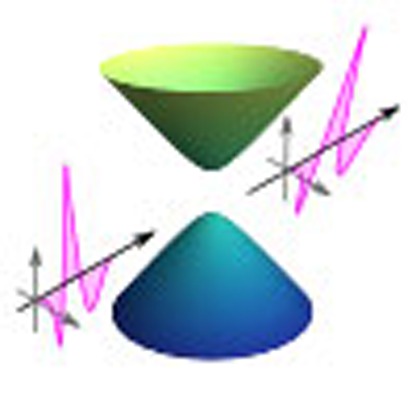

Topological quantum phenomena have been attracting increasing attention in condensed matter physics, because the system determined by the topological structure exhibits quantized observables, such as magnetic flux in superconductors and Hall conductance in quantum Hall effect. Magnetoelectric coupling, which has been a fundamental concept for contemporary physics including spintronics and multiferroics^1^, is predicted to be quantized in the recently discovered three-dimensional (3D) topological insulators (TIs)^2^,^3^,^4^,^5^,^6^,^7^,^8^,^9^,^10^. More specifically, quantized magnetoelectric responses are predicted on the quantum anomalous Hall (QAH) state induced by the magnetic mass-gap on the surface Dirac cone under the broken time-reversal symmetry. In the QAH state, as experimentally confirmed recently^11^,^12^,^13^,^14^,^15^,^16^,^17^,^18^, the surface states exhibit quantized Hall conductance (*σ*_*xy*_=*e*^2^*/h* and *σ*_*xx*_=0) without external magnetic field.

In the electromagnetic field in 3D TIs, the Lagrangian includes the axion term 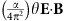, which is characterized by the fine structure constant 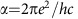 (refs 4, 19). Under the presence of time-reversal symmetry the term *θ* is equal to π in TI, while zero in the vacuum or ordinary insulator. When the time-reversal symmetry is broken at the TI surface, Maxwell's equations are modified, resulting in an unusual quantized magnetoelectric effect at the TI surface, referred to as topological magnetoelectric (TME) effect. This novel TME effect is ensured in a low-energy region below the magnetic mass gap at the Dirac point. Since the modified Maxwell's equations provide a quantized transverse current, the QAH effect with *σ*_*xy*_=*e*^2^/*h* can be viewed as a zero-frequency limit of TME effect. On the other hand, for the optical process the TME effect produces the quantized Faraday and Kerr rotation angles^4^,^6^,^7^, which represent polarization rotations for transmission and reflection geometries, respectively. Accordingly, the relation denoted with rotation angles (*θ*_F_ and *θ*_K_) always leads to the fine structure constant *α* (=2π*e*^2^/*hc*∼1/137), irrespective of material parameters such as dielectric constant and magnetic susceptibility, whereas the magneto-optical rotation angles of a thin film on substrate are substantially modified from those of the free-standing film in vacuum (*θ*_F_=*α*∼7.3 mrad and *θ*_K_=π/2 rad) (refs 4, 6, 7). Thus the observation of Faraday and Kerr rotations on QAH state provides a direct measure of *α*. As a result the rotation angles of electromagnetic wave in the low-energy region are scaled by the d.c. Hall conductance, in accord with the development of the QAH state. It should be emphasized that in spite of the identical origin, those two phenomena, QAH effect and topological Faraday and Kerr rotations, are observed as quantization of different physical quantities; *σ*_*xy*_=*e*^2^/*h* and *θ*_F_ =*α*=2π*e*^2^/*hc*. On the other hand, the difficulties in experimental verification of TME effect have been indicated since the early stage of theoretical predictions^4^,^6^,^7^. This is because the observation of TME effect requires the Fermi energy within the magnetic mass-gap on the surface Dirac cone, and hence precise Fermi energy tuning is indispensable. In addition, the observation of genuine TME signal is limited to the low energy, that is, sufficiently lower than the magnetic mass-gap to avoid the responses from real electronic transitions.

The QAH state on the surface of TI is stabilized by the magnetic mass-gap, while accurate size of the gap energy may depend on sample form (film/bulk) as well as concentration of magnetic dopants or defects. In fact, the gap energy has been reported to range from 22 meV for a Cr: Sb_2_Te_3_ thin film^20^ to 50 meV for a Cr: (Bi, Sb)_2_Te_3_ bulk single crystal^21^. Therefore, the magneto-optics by terahertz (THz) spectroscopy probing the lower-energy range, for example, 1–8 meV in the present experiment, is suitable for the observation of the possible emergence of topological Faraday/Kerr rotations. Furthermore, the recently developed magnetic modulation-doping in Cr: (Bi, Sb)_2_Te_3_ thin film^17^ can markedly widen the observable temperature region of QAH effect up to several Kelvin, making feasible the optical measurement of QAH state. So far the low-energy magneto-optical responses have been intensively studied^22^,^23^,^24^,^25^,^26^,^27^,^28^,^29^ mostly for nonmagnetic TIs, in which the cyclotron resonances of the surface states as well as the bulk carriers are reported. However, an experimental demonstration of TME effect on QAH state remains elusive.

In this paper, we show the experimental signature of topological Faraday/Kerr rotations in QAH states on magnetic TI thin films by THz time-domain spectroscopy (TDS). The trajectory towards the fine structure constant *α* is unveiled by the measurements of THz Faraday and Kerr rotation angles for the surface QAH state.

## Results

### QAH effect observed in a magnetic TI thin film

**Figure 1:**
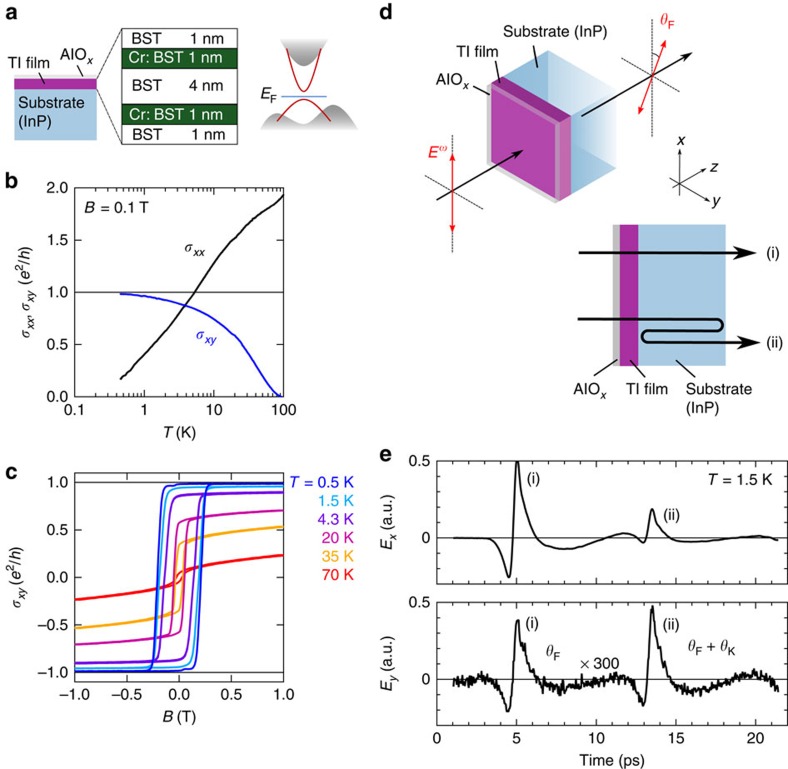
THz Faraday and Kerr rotation of QAH state on magnetic TI film. (**a**) Schematics of the TI film with magnetic modulation doping and of the band structure of surface states under the presence of time-reversal-symmetry breaking magnetization. (**b**) Temperature dependence of the longitudinal (*σ*_*xx*_) and Hall (*σ*_*xy*_) conductances at *B*=0.1 T. (**c**) Magnetic field dependence of *σ*_*xy*_ at various temperatures. (**d**) Schematics of the THz magneto-optics for the magnetic TI film on an InP substrate. Crossed-Nicol geometry was employed for the detection of the magneto-optical rotation of light polarization. Faraday and Kerr rotations are measured by the first THz pulse (i) and the second THz pulse (ii), respectively. (**e**) Time evolutions of THz pulses through the magnetic TI film at 0 T after the poling of magnetic moment. (See Methods for detail.) *E*_*x*_ and *E*_*y*_ are transmitted light polarized parallel and perpendicular to the incident light, respectively. The first pulse (i) represents the directly transmitted light through the TI film and substrate, while the THz pulse once reflected at back surface of substrate appears as the second pulse (ii) with a time delay. *E*_*y*_ component of the second pulse includes the Kerr rotation *θ*_K_ at back-surface of TI film as well as the Faraday rotation *θ*_F_.

The Cr_*x*_(Bi_0.26_Sb_0.74_)_2−*x*_Te_3_ TI film with magnetic modulation-doping^17^, where magnetic impurities Cr (*x*=0.57) are doped in two quintuple layers adjacent to the top and bottom (Bi_0.26_Sb_0.74_)_2_Te_3_ layers^17^, is schematically illustrated in Fig. 1a. The evolution of the magnetization induced by the Cr-doping gives rise to the QAH state as shown in Fig. 1b,c. The ferromagnetic transition occurs around *T*_C_∼70 K with the onset of the anomalous Hall term in *σ*_*xy*_ (Fig. 1b). As temperature decreases, *σ*_*xy*_ develops and tends to saturate at the quantized value *e*^2^/*h* at around *T*=0.5 K, while *σ*_*xx*_ steeply decreases towards zero, due to the emergence of the dissipationless chiral edge conduction (Fig. 1b). The Hall angle (*σ*_*xy*_*/σ*_*xx*_) becomes as large as 1 around 4 K, indicating the emergence of QAH regime at temperatures more than an order of magnitude higher^17^ than the uniformly Cr- or V-doped TI films^11^,^12^,^13^,^14^,^15^,^16^,^18^, due perhaps to the enlargement of the magnetic mass-gap induced by the rich Cr-doping and the reduced disorder in the surface states by Cr dopants^17^. Hysteretic behaviours of Hall conductance further evidence the development of the QAH regime as shown in Fig. 1c. The fully quantized *σ*_*xy*_ at the lowest temperature indicates that the Fermi energy locates well within the magnetic mass-gap of the surface Dirac cone (Fig. 1a) without additional field-effect tuning.

### THz magneto-optics on the magnetic TI thin film

THz-TDS provides magneto-optical measurements with sufficiently lower photon energy (1–8 meV) than the magnetic mass-gap (20–50 meV (refs 20, 21)) and with high resolution of light-polarization rotations (<1 mrad). Recently, this technique has been found to be useful to study polarization rotation in THz region on ferromagnetic semiconductors as well^30^. The measurement configuration of magneto-optics by THz-TDS is schematically illustrated in Fig. 1d (see Methods for detail). Depending on the time delay, the monocycle THz pulse can differentiate the directly transmitted pulse (i) and the delayed pulse generated by back-reflection at the back surface of substrate (ii), as shown in Fig. 1d,e; this enables us to separably measure Faraday and Kerr rotations, as reported for TI thin films^22^,^24^ and graphene on substrates^31^. As shown in Fig. 1e, the temporal waveform of *E*_*y*_-component indicates the pronounced rotation of polarization on the first pulse (i) as well as on the second one (ii) due to the presence of the magneto-optical rotations at zero external magnetic field. The first pulse (i) involves the Faraday rotation (*θ*_F_), while the second pulse (ii) is composed of *θ*_F_ plus the Kerr rotation (*θ*_K_) at the back surface of the magnetic TI film (Fig. 1d).

**Figure 2:**
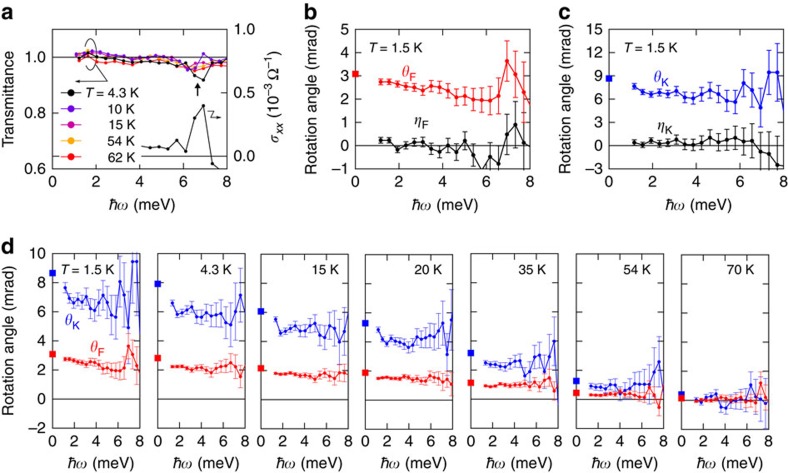
THz Faraday and Kerr rotation spectra of the magnetic TI film. (**a**) Transmittance (left ordinate) spectra of the magnetic TI film at various temperatures and optical conductance (right ordinate) *σ*_*xx*_ spectrum at *T*=4.3 K. The transmittance close to unity was observed, indicating the negligible carrier absorptions due to fine-tuning of the Fermi level within the magnetic mass-gap of the surface state. The low-lying optical phonon mode is discerned as a tiny dip around 7 meV and also as the peak of optical conductance spectrum. (**b**,**c**) Complex Faraday (**b**) and Kerr (**c**) rotation spectra at 1.5 K (see the main text and Methods for definition). The real parts (*θ*_F_ and *θ*_K_) represent the rotation angle of light polarization. The imaginary parts (*η*_F_ and *η*_K_) represent the ellipticity, which is negligibly small as expected. Rotation angles at *ω*=0 evaluated from the d.c. transport data (Fig. 1c) are also plotted on the left ordinates. (**d**) Temperature dependence of the Faraday (red) and Kerr (blue) rotation spectra with the evaluated *ω*=0 values on the ordinates. The error bars in **b**–**d** are evaluated by s.e.m. of several runs of measurement.

The transmittance spectra obtained by *E*_*x*_-component at different temperatures are shown in Fig. 2a. The transmittance is close to unity, that is, no discernible absorption, except for the dips around 7 meV indicated by the arrow, which is assigned to the optical phonon mode^32^; see also the optical conductance *σ*_*xx*_ spectrum at *T*=4.3 K also shown in Fig. 2a. The negligibly weak absorption, for example, no Drude response, confirms that the Fermi energy locates within the magnetic mass-gap of the surface Dirac cone (Fig. 1a).

Fourier transformation of the electric field pulses *E*_*x*_(*t*) and *E*_*y*_(*t*) (Fig. 1e) provides the complex Faraday and Kerr rotation spectra (Fig. 2b,c), where the real part, *θ*_F_(*ω*) or *θ*_K_(*ω*), and the imaginary part, *η*_F_(*ω*) or *η*_K_(*ω*), represent the rotation angle and the ellipticity, respectively (see Methods for detail). The rotation-angle (real part) spectra for *θ*_F_ and *θ*_K_ show finite values around 2.6 and 6.9 mrad, respectively, with modest frequency dependence, as shown in Fig. 2b,c. The ellipticity (imaginary part) spectra for *η*_F_ and *η*_K_ are close to zero in the whole photon-energy region (<8 meV). Note that noise-like fringe structures in the Kerr rotation spectra (Fig. 2c,d), in contrast to the almost *ω*-constant Faraday rotation spectra, come from the inevitable interference due to the temporal overlap with the early-coming Faraday rotation signals (Fig. 1e). These characters, that is, little frequency dependence and near-zero ellipticity, strongly indicate that the current THz energy window (1–8 meV) is well below the threshold energy for any magneto-optically active real transitions. This is consistent with the fact that the magnetic mass-gap on the Dirac point (reported to be 20–50 meV by scanning tunnel spectroscopy^20^,^21^) is sufficiently large as compared with the energy range of this measurement. Note also that possible cyclotron resonance under magnetic field, which has been observed in previous magneto-optical studies on TIs^22^,^23^,^24^,^25^,^26^,^27^,^28^, is absent in the present measurement because of zero external magnetic field. Furthermore, the observed Faraday and Kerr rotation angles are quantitatively consistent with the estimated rotation angles at d.c. limit (Fig. 2b,c), which are calculated from *σ*_*xx*_ and *σ*_*xy*_ obtained by the d.c. transport measurement (Fig. 1c) through the following relations^26^,^31^;


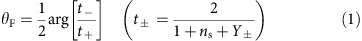



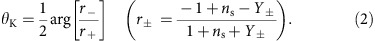


**Figure 3:**
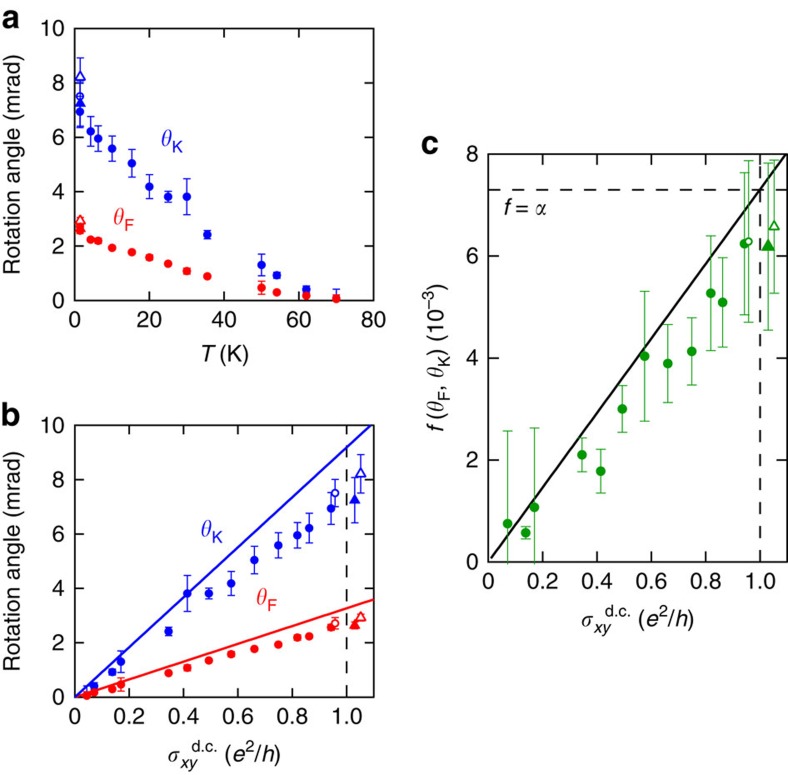
Trajectory towards the quantized TME response. Closed circles correspond to the rotation spectra in Fig. 2b–d. Closed triangles represent the data of another different sample. Open symbols represent the data at *B*=1 T. (**a**,**b**) Faraday (red) and Kerr (blue) rotation angles versus temperature (**a**) and d.c. Hall conductance *σ*_*xy*_^d.c.^ (Fig. 1c) (**b**). The rotation angles and their error bars in **a**,**b** are determined by the mean and s.d. of the rotation angle spectra below∼4 meV, respectively. The solid lines in **b** represent the estimation from equations (1) and (2) in the main text. (**c**) Evolution of the scaling function 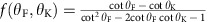 (see the main text and equation (3)) without any material parameters as a function of d.c. Hall conductance, which is expected to reach the fine structure constant *α* (=2π*e*^2^/*hc*∼1/137) in the quantized limit, as indicated by a straight line. The error bars of *f* (*θ*_F_, *θ*_K_) are determined by its total derivative calculated from s.d. of *θ*_F_ and *θ*_K_ presented in **a**,**b**.

Here *t*_+(−)_ and *r*_+(−)_ represent the transmittance and reflection coefficients of right- and left-handed circularly polarized light, respectively. The admittance *Y*_±_ is described as *Y*_±_=*Z*_0_(*σ*_*xx*_±i*σ*_*xy*_) (*Z*_0_=377 Ω: the vacuum impedance) and *n*_s_ is the refractive index of the InP substrate. We determined *n*_s_ as 3.47 by measuring THz response of the substrate, which well agrees with literature^33^. For instance, the estimated rotation angles for *θ*_F_ and *θ*_K_ at *ω*=0 are around 3.1 and 8.7 mrad at 1.5 K (indicated with closed squares on the respective ordinates in Fig. 2b,c). This quantitative agreement with the d.c. QAH state exemplifies that the observed THz rotation stems from the TME effect on the TI surfaces. Figure 2d shows the temperature evolution of the Faraday and Kerr rotation spectra. The rotation angles decrease with increasing temperature and vanish at *T*_C_ (see also Fig. 3a), in accord with the disappearance of the ferromagnetic state. We also confirmed that the Faraday and Kerr rotations arising from the QAH state are reproducibly observed for the different sample with different heterostructure and *T*_C_ (∼40 K) (see Supplementary Figs 1 and 2 and Supplementary Note 1).

Since the magnetic-layer thickness in the present heterostructure film is 2 nm in total, the figure of merit of spontaneous Faraday rotation of our sample can be effectively regarded as ∼7 × 10^5^ degree per cm in the off-resonant condition with near-zero ellipticity. For comparison, Faraday rotation of most well-known Faraday rotator Bi-doped yttrium iron garnet is 9 × 10^2^ degree per cm around 1 eV (ref. 34). Thus the rotation angle observed here is remarkably large as compared with the conventional Faraday rotation in ferromagnets. Furthermore, we observed the nearly same magnitude of rotation angles on the different heterostructure TI sample with twice the thickness of magnetic layer (4 nm in total) (see Supplementary Figs 1 and 2 and Supplementary Note 1). These results strongly indicate that the polarization rotations observed here intrinsically originate from the response of the TI surface states.

### Trajectory towards topological Faraday and Kerr rotations

In Fig. 3b the rotation angles at different temperatures, which are measured by averaging the rotation angle below ∼4 meV (Fig. 2d), are displayed (closed circles) as a function of the d.c. Hall conductance together with the calculated values from equations (1) and (2) at *ω*=0. Note that open circles in Fig. 3 correspond to the rotation angles measured at *B*=1 T (see Supplementary Fig. 3 and Supplementary Note 2). We also plot the data for the different sample at *T*=1.5 K and *B*=0 T (1 T) denoted by closed (open) triangles (see Supplementary Figs 1 and 2 and Supplementary Note 1). The observed THz Faraday and Kerr rotation angles show a good agreement with the estimated *ω*=0 value, although small deviations are still discerned.

The relationship between the Faraday and Kerr rotation angles at the quantized limit is expected to lead to the fine structure constant *α*, irrespective of any material parameters such as the dielectric constant and the thickness of the film, the capping layer and the substrate^6^. In our measurement geometry, the universal relationship between *θ*_F_ and *θ*_K_ in the QAH state is obtained from equations (1) and (2);


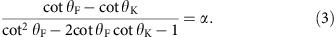


Here we define the left side of equation (3) as the scaling function *f* (*θ*_F_, *θ*_K_). In Fig. 3c, the function *f* (*θ*_F_, *θ*_K_) versus d.c. Hall conductance *σ*_*xy*_^d.c.^ is plotted, in which the *f* (*θ*_F_, *θ*_K_) is expected to reach *α* (=2π*e*^2^/*hc*∼1/137) in the quantized limit. With increasing *σ*_*xy*_ to the quantized conductance by lowering temperature, the dimensionless *f* (*θ*_F_, *θ*_K_) approaches the universal value *α*, in good agreement with the estimation at *ω*=0 (line in Fig. 3c), manifesting the trajectory towards the quantized value *α* determined uniquely and solely by the magneto-optical rotation angles.

The small deviation in *θ*_F_, *θ*_K_ and *f* (*θ*_F_, *θ*_K_) from the estimation based on d.c. Hall conductance are discerned (Fig. 3b,c). One reason for the small reduction of the rotation angles from the d.c. limit may be partial magnetization reversal under zero magnetic field during the terahertz measurements (Fig. 1e). Indeed, the measurements under magnetic field of *B*=1 T, where the magnetization reversal is totally avoided during the measurement, result in slightly higher values of Faraday and Kerr rotations and hence of *f* (*θ*_F_, *θ*_K_) (open circles and triangles in Fig. 3b,c), although slight deviations from the expectations are still discerned. Another possible cause is a difference of the characters between the d.c. Hall measurement and optical one in the quantum Hall regime. The d.c. Hall measurement detects the conduction of the chiral edges states developing at the sample edge, irrespective of inside small domains or islands where the quantization may remain incomplete due to defects with potential hills/valleys or to residual in-gap states^35^. On the other hand, since optical measurement detects the conductance averaged over the spot area, the obtained rotation angles might be reduced from the d.c. limit due to those islands. A certain amount of residual d.c. longitudinal conductance (*σ*_*xx*_) at the lowest temperature (1.5 K) of the present optical experiment implies the persistence of such an effect (Fig. 1b), which would be thoroughly cleared up at further lower temperatures where *σ*_*xx*_∼0 is attained.

After submitting the manuscript, two other groups uploaded preprints on an e-Print server reporting quantized magneto-optical rotations, which were observed on nonmagnetic TIs under application of magnetic field^36^,^37^. In the present study on the quantum anomalous Hall effect in zero magnetic field, we have observed the almost quantized Faraday and Kerr rotations via exchange interaction with localized magnetic moments instead of external magnetic field, that is, without any contribution from the cyclotron motion of conduction electron.

In conclusion, we have experimentally investigated the TME effect on the QAH state of surface state of TI by measurements of Faraday and Kerr rotations in THz region. The observed Faraday and Kerr rotation angles show quantitative agreement with the estimation from the d.c. transport results. The universal relationship with the magneto-optical rotation angles shows the trajectory converging to the fine structure constant *α* with the approach to the QAH state.

## Methods

### Sample fabrication

The 8-nm-thick TI films with magnetic modulation doping were grown on both-side-polished insulating InP substrates by molecular beam epitaxial growth as described in ref. 17. To protect the film from degradation, a 3-nm-thick AlO_*x*_ layer was immediately deposited with *ex situ* atomic layer deposition. Transport measurement and optical THz spectroscopy were performed on different samples from the same batch for each film. Possible modification of rotation angles by the AlO_*x*_ capping layer is estimated to be as small as 0.01% at most, and hence neglected in the analysis described in the main text.

### Magneto-optical terahertz spectroscopy

In the THz-TDS, laser pulses with duration of 100 fs from a mode-locked Ti: sapphire laser were split into two paths to generate and detect THz pulses. THz pulses were generated by a bow-tie-shaped antenna and detected by a dipole antenna. The *E*_*y*_(*t*) component of the transmitted THz pulses (Fig. 1e) was measured by the Crossed-Nicole configuration by using wire-grid polarizers. The polarization rotation *E*_*y*_(*t*) at 0 T is defined by the anti-symmetrized waveform to eliminate the background signal, which is the difference between signals with magnetization for ±*z* directions after the poling of the magnetization at ±1 T; 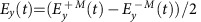. The Faraday rotation of the substrate (InP) is smaller than the sensitivity of our equipment (<10 μrad T^−1^). The Fourier transformation of the first THz pulses *E*_*x*_(*t*) and *E*_*y*_(*t*) (Fig. 1e) gives the complex Faraday rotation spectra *E*_*y*_(*ω*)/*E*_*x*_(*ω*)=(sin*θ*_F_(*ω*)+i*η*_F_(*ω*)cos*θ*_F_(*ω*))/(cos*θ*_F_(*ω*)−i*η*_F_(*ω*)sin*θ*_F_(*ω*))∼*θ*_F_(*ω*)+i*η*_F_(*ω*) (Fig. 1b) for the small rotation angles. The rotation spectra obtained by the second pulses give the sum of the Kerr and Faraday rotation spectra. In Kerr rotation spectra, inevitable interference with early-coming Faraday rotation signal in time domain (Fig. 1e) results in the fringe structures. We carefully examined the interference in the raw data and extracted Kerr signal to minimize them. Transmittance spectra were obtained by comparison between the transmission of sample and bare substrate. We applied the following standard formula to obtain the complex conductance *σ*(*ω*)=*σ*_1_(*ω*)+i*σ*_2_(*ω*) of TI film;


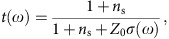


where *t* (*ω*) is the complex transmittance, *Z*_0_ is the impedance of free space (377 Ω) and *n*_s_ the refractive index of the InP substrate.

### Data availability

The authors declare that the data supporting the findings of this study are available within the article and its Supplementary Information.

## Additional information

**How to cite this article:** Okada, K. N. *et al*. Terahertz spectroscopy on Faraday and Kerr rotations in a quantum anomalous Hall state. *Nat. Commun.* 7:12245 doi: 10.1038/ncomms12245 (2016).

## Supplementary Material

Supplementary Information Supplementary Figures 1-3, Supplementary Notes 1-2
