# Supplementary material for: Terahertz spectroscopy on Faraday and Kerr rotations in a quantum anomalous Hall state
Source: Nat Commun. 2016 Jul 20;7:12245. doi: 10.1038/ncomms12245 (PMC4961790; doi:10.1038/ncomms12245)
Supplement: Supplementary Information — Supplementary Figures 1-3, Supplementary Notes 1-2 [file ncomms12245-s1.pdf]

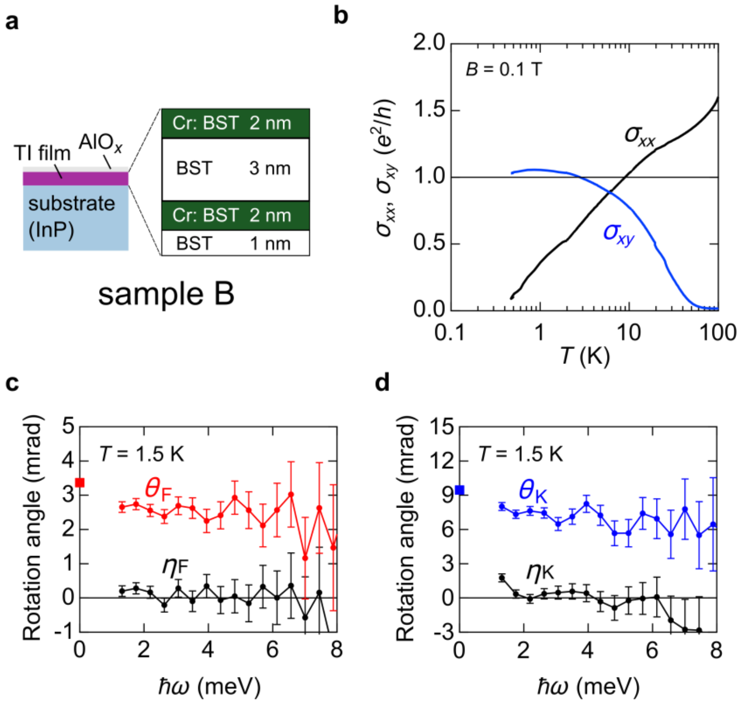

Supplementary Figure 1| Transport and magneto-optical properties in another magnetic TI film. (**a**) Schematic structure of the TI film (sample B) with different magnetic modulation doping from that (sample A, Fig. 1a) presented in main text. (**b**) Temperature dependence of σ_{xx} and σ_{xy} at B = 0.1 T. (**c**,**d**) Complex Faraday (**c**) and Kerr (**d**) rotation spectra at T = 1.5 K at zero magnetic field. The real parts (θ_F and θ_K) represent the rotation angle of light polarization. The imaginary parts (η_F and η_K) represent the ellipticity. The error bars in **c** and **d** are evaluated by s.e.m. of several runs of measurement.

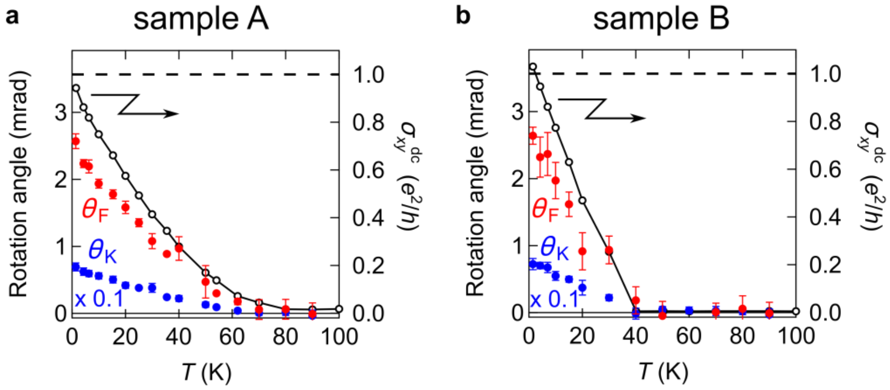

Supplementary Figure 2| Comparison of temperature evolution of magneto-optical rotation angles for sample A and B. (**a**,**b**) Temperature dependence of Faraday and Ker rotation angles, together with $\sigma_{xy}{}^{dc}$ (right ordinate) on the magnetic TI film for the sample A (Fig. 1a) (**a**) and the sample B (Supplementary Fig. 1a) (**b**). The rotation angles and their error bars in **a** and **b** are determined by the mean and s.d. of the rotation angle spectra below ~ 4 meV, respectively. The right ordinate for $\sigma_{xy}{}^{dc}$ is correctly scaled to the left ordinate θ_F via the relation of Eq. (1) in main text.

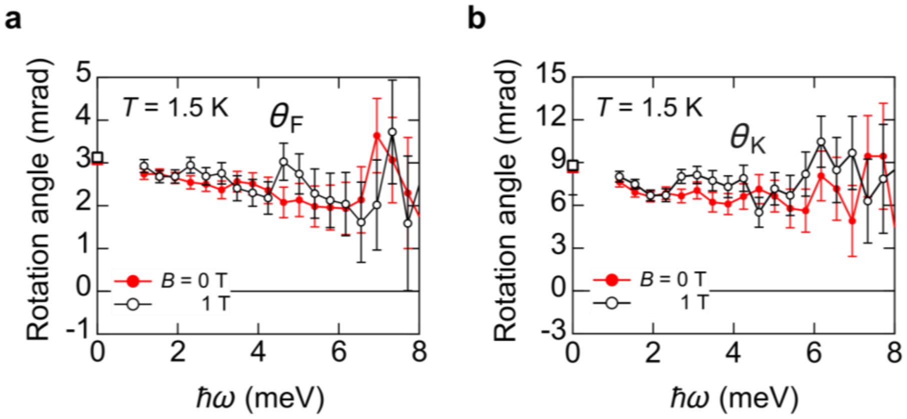

Supplementary Figure 3| Magneto-optical rotation spectra under application of 1 T. (**a**,**b**) Faraday (θ_F) (**a**) and Kerr (θ_K) (**b**) rotation angle spectra at T = 1.5 K under B = 0 T and 1 T, measured on the sample A (Fig. 1a) presented in the main text. The error bars are evaluated by s.e.m. of several runs of measurement.

Supplementary Note 1| Observation of Faraday and Kerr rotations on different magnetic topological insulator (TI) film

We prepared a different TI sample (referred to as sample B) from that (sample A) presented in main text (Fig. 1a), which has a similar type of magnetic modulation-doping but with a different heterostructure (Supplementary Fig. 1a). The Hall angle (σ_{xy}/σ_{xx}) exceeds 1 below 6 K (Supplementary Fig. 1b), indicating the presence of QAH state on this sample, while T_C is suppressed (~40 K, see Supplementary Fig. 2b) compared with the sample A (T_C ~ 70 K). The complex Faraday and Kerr rotation spectra at T = 1.5 K for the sample B are shown in Supplementary Figs. 1c and 1d, respectively. The flat rotation angle spectra and near-zero ellipticity indicate that the frequency range of our experiment is well below any magneto-optically

active excitations, whereas T_C is suppressed. The Faraday and Kerr rotation angles for the sample B also show good agreement with dc values (closed squares on the ordinates in Supplementary Figs. 1c and 1d) calculated through Eq. (3) in the main text.

In Supplementary Fig. 2 we compare the temperature evolution of θ_F and θ_K, together with dc Hall conductance (right ordinate) for the both TI films (sample A and B). Note that the right ordinate for σ_{xy} is scaled to the left ordinate θ_F via the relation of Eq. (1) in main text. Supplementary Fig. 2 indicates that T_C is largely different; T_C ~ 70K for the sample A in the main text (Supplementary Fig. 2a), while T_C ~ 40 K for the sample B (Supplementary Fig. 2b). In the both samples Faraday and Kerr rotations arise at around T_C and develop in accord with σ_{xy}^{dc}.

Supplementary Note 2| Magneto-optical rotation spectra under magnetic field

At T = 1.5 K we performed the magneto-optical measurement at 1 T. Faraday and Kerr rotation spectra for the sample A (Fig. 1a) are shown in Supplementary Figs. 3a and 3b. The rotation spectra under B = 1 T are shown together with those under B = 0 T, the both being consistent with the dc values (see open (1 T) and closed (0 T) squares on the ordinate in Supplementary Fig. 3). The averages of the rotation angles below 4 meV result in slightly higher values for B = 1 T (see Figs. 3a and 3b in the main text), probably because of the absence of the reversal of ferromagnetic domain. Note that the Faraday rotation of the substrate (InP) is smaller than the sensitivity of our equipment (< 10 μrad T^{-1}).
